# Effects of Permissive Hypercapnia Versus Normocapnia on Cerebral Oxygenation and Postoperative Outcomes in Adult Surgical Patients: A Systematic Review and Meta‐Analysis

**DOI:** 10.1155/anrp/3569464

**Published:** 2026-10-31

**Authors:** Areej Riaz, Amna Khan, Fatima Nawaz, Amna Shafaqat, Hadia Sheraz, Mohammed M. AlTelbany, Nauroze Khan, Adeel Ahmed Khan, Bismah Rafique Malik, Hermann Yokolo

**Affiliations:** ^1^ Department of Medicine Services Institute of Medical Sciences Lahore Pakistan sims.edu.pk; ^2^ Department of Medicine Ameer-ud-Din Medical College Lahore Pakistan duhs.edu.pk; ^3^ Department of Medicine University of Health Sciences Lahore Pakistan uhs.edu.kh; ^4^ Department of Medicine FMH College of Medicine and Dentistry Lahore Pakistan duhs.edu.pk; ^5^ Faculty of Medicine Al-Azhar University Gaza State of Palestine azhar.edu.eg; ^6^ Department of Medicine Pakistan Institute of Medical Sciences Islamabad Pakistan pims.gov.pk; ^7^ Department of Medicine Sindh Government Hospital Karachi Pakistan duhs.edu.pk; ^8^ Department of Medicine Dow University of Health Sciences Karachi Pakistan duhs.edu.pk; ^9^ Department of Research Medical Research Circle (MedReC) Goma Democratic Republic of the Congo

**Keywords:** hypercapnia, intraoperative cerebral oxygenation, meta-analysis, postoperative cognitive dysfunction, postoperative delirium, surgery

## Abstract

**Background:**

Evidence suggests that intraoperative cerebral hypoxia is the main contributor of postoperative neurological complications, particularly postoperative cognitive dysfunction (POCD) and postoperative delirium (POD) in surgical patients. Permissive hypercapnia may improve cerebral oxygenation by increasing cerebral blood flow, but its perioperative benefits remain uncertain.

**Research Question:**

In adult surgical patients, is permissive hypercapnia associated with improved cerebral oxygenation and perioperative outcomes compared with normocapnia?

**Method:**

PubMed, Embase, Cochrane CENTRAL, and ClinicalTrials.gov were searched from inception to April 2026 for randomized controlled trials (RCTs) and cohort studies of adult surgical patients receiving permissive hypercapnia. Primary outcome included was intraoperative regional cerebral oxygen saturation (rSO_2_). Secondary outcomes were POCD, POD, all‐cause mortality, respiratory parameters, arterial blood gas variables, and hemodynamic outcomes.

**Results:**

Seven studies comprising 824 patients were included. Permissive hypercapnia significantly improved intraoperative rSO_2_ (MD = 6.65%; 95% Cl: [4.93, 8.37]; *p* < 0.00001), PaCO_2_ (MD = 10.21 mmHg (95% CI: [8.81, 11.60]; *p* < 0.00001), and end‐tidal CO_2_ (MD = 10.83 mmHg; 95% CI [7.91, 13.76]; *p* < 0.00001), and significantly reduced dead space fraction (MD = −6.38%; 95% CI: [–9.26, −3.50], *p* < 0.00001), and arterial pH (MD = −0.08; 95% CI: [‐0.09, −0.07]; *p* < 0.00001). Hypercapnia had no significant effect on PaO_2_, mean arterial pressure, and heart rate. Qualitative analysis suggested reduced incidence of POCD with no significant effect on POD.

**Conclusion:**

Permissive hypercapnia improves intraoperative cerebral oxygenation while maintaining hemodynamic stability. However, the available evidence for POCD and POD is limited and heterogeneous. Larger RCTs and observational studies are needed to confirm its effects on clinical outcomes before routine implementation.

## 1. Introduction

Surgical procedures are increasingly performed worldwide, exposing patients to potential cerebral hypoxia, particularly affecting elderly patients [1]. When cerebral oxygen levels drop during surgery, patients may develop postoperative cognitive dysfunction (POCD), which seriously impacts patients’ quality of life and prognosis [2, 3]. However, permissive hypercapnia, a ventilation strategy that can tolerate mild CO_2_ accumulation, has been shown to improve systemic circulation and cerebral blood flow (CBF) [4, 5]. This effect is achieved by CO_2_‐induced cerebral vasodilation, which may reduce the risk of cerebral hypoxia and help maintain cerebral oxygenation [6].

Although some studies have investigated pH in surgical settings, the available evidence is limited to specific surgical categories and did not provide definitive evidence [1, 6, 7]. Therefore, a pooled analysis is needed to combine all available evidence across different surgical procedures and populations.

Thus, this systematic review and meta‐analysis aims to evaluate the effects of permissive hypercapnia compared with normocapnia on rSO_2_, POCD, POD, arterial pH, respiratory and hemodynamic parameters in adults undergoing surgery.

## 2. Methodology

### 2.1. Study Design

The study was conducted according to the Preferred Reporting Items for Systematic Reviews and Meta‐Analyses (PRISMA) guidelines. The study protocol was registered prospectively on International Prospective Register of Systematic Reviews (PROSPERO; CRD420261449137). Ethical approval was not required as we only used publicly available data (Figure 1).

**FIGURE 1:**
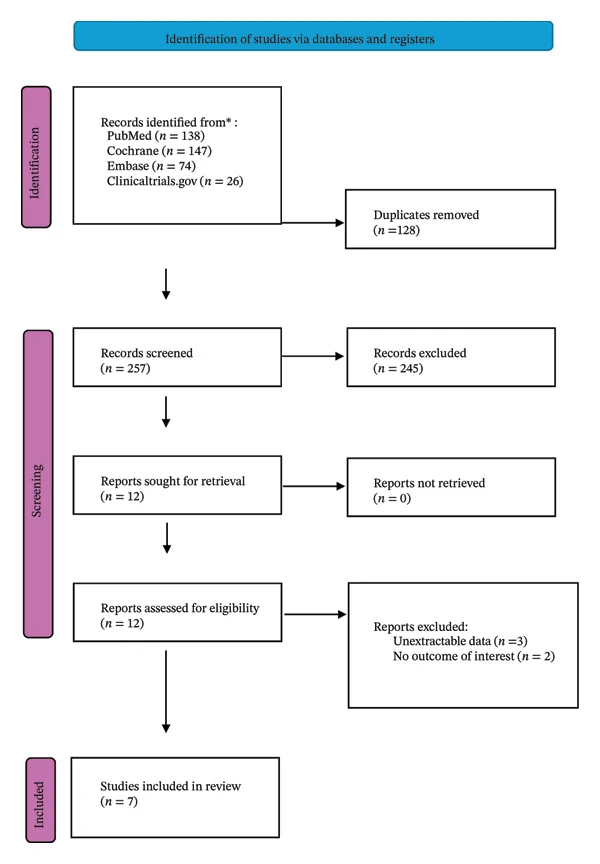
PRISMA flowchart.

### 2.2. Data Sources and Search Strategy

We conducted a comprehensive search of EMBASE, PubMed, the Cochrane Central Register of Controlled Trials (CENTRAL), and ClinicalTrials.gov from inception to April 2026 for RCTs and cohorts assessing the effects of permissive hypercapnia in diverse surgical patients. The detailed search string can be assessed in Supporting File 1.

### 2.3. Study Selection

A predefined PICOS framework was used to determine study eligibility.

 Population: Adults (≥ 18 years) undergoing any surgical procedure.
 Intervention: Permissive intraoperative hypercapnia.
 Comparator: Normocapnia.
 Outcomes: rSO_2_, POCD, POD, respiratory and hemodynamic parameters.

Studies including pediatric populations, no comparator arm, review articles, abstracts, editorials, and articles with insufficient or unavailable data were excluded.

### 2.4. Primary and Secondary Outcomes

The predefined primary outcome was rSO_2_ level measured using near‐infrared spectroscopy (NIRS). The secondary outcomes were incidence of POCD and POD, all‐cause mortality, arterial pH respiratory, and gas exchange parameters including partial pressure of arterial carbon dioxide (PaCO_2_), end‐tidal carbon dioxide (ETCO_2_), partial pressure of arterial oxygen (PaO_2_), dead space fraction (Vd/Vt), and hemodynamic parameters including mean arterial pressure (MAP) and heart rate (HR).

### 2.5. Data Extraction

Data were extracted independently by two reviewers using an Excel spreadsheet. Extracted study characteristics included first author, year of publication, country, study design, type of surgery, sample size, and baseline patient characteristics. For all primary and secondary outcomes, raw event counts or mean values with standard deviation (SD) were extracted.

### 2.6. Statistical Analysis

Statistical analysis was performed using Review Manager (RevMan, Version 5.4). For continuous variables, mean differences (MDs) with 95% confidence intervals (CIs) were calculated. For dichotomous variables, risk ratios (RRs) were calculated using the inverse variance model to provide the estimates. Statistical heterogeneity was evaluated using Cochran’s Q test and I^2^ statistic. Heterogeneity was interpreted as low (< 25%), moderate (25%–50%), substantial (50%–75%), and considerable (> 75%). A *p* value less than 0.05 was considered statistically significant.

### 2.7. Risk of Bias Assessment

The risk of bias of the included RCTs was assessed using the Cochrane Risk of Bias 2 (RoB 2) tool. For the observational studies, ROBINS‐I tool was used.

### 2.8. Certainty of Evidence

The certainty of evidence for each outcome was determined using the GRADE framework [8]. Certainty was assessed based on risk of bias, inconsistency, indirectness, imprecision, and publication bias. The certainty of evidence was rated as high, moderate, low, or very low.

## 3. Results

A total of 385 records were initially obtained from the database search including 138 from PubMed, 147 from Cochrane CENTRAL, 74 from Embase, and 26 from ClinicalTrials.gov. After removing 128 duplicates using Endnote, two authors independently screened the records using Rayyan software. Discrepancies were resolved through mutual discussion or through consultation with a third investigator. After removing ineligible studies, the full text of 15 studies was assessed. Of these, seven studies, including six RCTs and one observational cohort, were finally included in the analysis [1, 7, 9–13].

### 3.1. Baseline Characteristics

Seven studies comprising 412 patients receiving permissive hypercapnia and 412 patients receiving normocapnia were included in the meta‐analysis [1, 7, 9–13]. Across the included studies, mean age ranged from 48 to 68 years. Baseline demographic and clinical characteristics were generally comparable between the hypercapnia and normocapnia groups within individual studies (Table 1).

**TABLE 1:** Baseline characteristics.

Variable	Zhu et al. [13]		Akça et al. [9]	Kwak et al. [11]		Bhandari et al. [10]		Chen et al. [7]		Lv et al. [1]		Park et al. [12]	
	Normocapnia (*n* = 33)	Hypercapnia (*n* = 33)	Overall (*n* = 10)	Normocapnia (*n* = 20)	Hypercapnia (*n* = 20)	Hypercapnia (*n* = 50)	Normocapnia (*n* = 50)	Hypercapnia (*n* = 225)	Normocapnia (*n* = 225)	Normocapnia (*n* = 36)	Hypercapnia (*n* = 36)	Normocapnia (*n* = 28)	Hypercapnia (*n* = 29)
Age (years)	60.27 (9.19)	61.52 (7.97)	56 (42–62)	53 (8)	50 (9)	60.73 (7.75)	61.54 (8.06)	68.21 (7.06)	67.95 (8.11)	58.0 (10.2)	55.5 (11.2)	48 ± 5	48 ± 7
Sex (M/F)	—	—	3/7	10/10	13/7	33/17	39/11	118/107	120/105	24/12	29/7	—	—
Body weight (kg)	63.94 (11.46)	64.32 (12.08)	84 (21)	64 (10)	69 (10)	64.66 (12.07)	67.19 (11.12)	—	—	—	—	—	—
BMI (kg/m^2^)	—	—	28 (5)	—	—	—	—	27.01 (2.65)	26.84 (2.77)	22.2 (2.6)	23.2 (2.5)	24 ± 3	25 ± 3
Hemoglobin, mg/dL	—	—	14 (2)	—	—	12.79 (1.61)	12.76 (1.63)	—	—	—	—	—	—
Pre‐op Hb (g/dL)	—	—	—	—	—	—	—	—	—	13.1 (1.5)	13.1 (1.6)	—	—
Post‐op Hb (g/dL)	—	—	—	—	—	—	—	—	—	12.2 (1.3)	12.3 (1.6)	—	—
ASA status I/II	—	—	—	—	—	—	—	212 (94.22)	210 (93.33)	8 (22.2)	10 (27.8)	—	—
ASA status III	—	—	10 (100)	—	—	—	—	13 (5.78)	15 (6.67)	—	—	—	—
ASA status IV	—	—	—	—	—	—	—	—	—	—	—	—	—
Surgery duration (min)	227.42 (52.56)	221.21 (43.61)	203 (38)	—	—	—	—	—	—	238.2 ± 89.5	217.4 ± 82.1	65 ± 30	—

*Note:* F, female; M, male; Hb, hemoglobin; Pre‐op, preoperative; Post‐op, postoperative.

Abbreviations: ASA, American Society of Anesthesiologists; BMI, body mass index.

### 3.2. Primary Outcome

#### 3.2.1. Intraoperative Regional Cerebral Oxygenation (rSO_2_)

Four trials, comprising 608 patients provided quantitative data regarding rSO_2_ [1, 7, 9, 13]. Random‐effects meta‐analysis revealed that mild hypercapnia significantly increased intraoperative rSO_2_ compared with normocapnia, showing a pooled MD of 6.65% (95% CI: [4.93, 8.37]; *p* < 0.00001). Moderate statistical heterogeneity was observed across the trials (*I* ^2^ = 36%). To explore the source of heterogeneity, a leave‐one‐out sensitivity analysis was performed, which showed that excluding Chen et al. [7] eliminated heterogeneity (Figure 2 and Supporting Figure 1).

**FIGURE 2:**
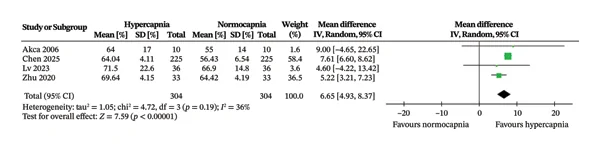
Forest plot comparing the effect of mild hypercapnia versus normocapnia on intraoperative regional cerebral oxygen saturation (rSO_2_).

### 3.3. Secondary Outcome

#### 3.3.1. POCD and Postoperative Delirium

A qualitative narrative synthesis was performed to evaluate the occurrence of POCD and POD due to differences in follow‐up period and assessment tools. Three studies evaluated POCD using the Mini‐Mental State Examination (MMSE) or its standardized version (SMMSE), and two studies reported the incidence of POD. Overall, the available evidence suggested better cognitive performance in patients receiving permissive hypercapnia. In the retrospective cohort by Chen et al., MMSE scores were significantly higher in patients receiving hypercapnia than those in the normocapnia group on postoperative days 1, 7, and 14 [7]. However, mean MMSE scores remained within the range of mild cognitive impairment. Zhu et al. also reported that patients receiving mild hypercapnia had significantly higher MMSE scores and a lower incidence of POCD at 24 h than those receiving normocapnia [13]. In the randomized trial by Bhandari et al. assessing POCD using SMMSE scores, mild hypercapnia demonstrated better preservation of cognitive function during the early postoperative period [10].

Evidence regarding POD was mixed. Lv et al. reported no significant difference in the occurrence of POD between the treatment groups [1]. Park et al., on the other hand, observed no episodes of POD among study participants in either of the groups [12].

#### 3.3.2. Partial Pressure of Arterial Carbon Dioxide (PaCO_2_)

Six trials reported PaCO_2_ (mmHg) levels across 713 patients [7, 9–13]. The random‐effects model showed a significantly higher PaCO_2_ levels in the intervention arm than in the comparator arm (MD = 10.21 mmHg; 95% CI: [8.81, 11.60]; *p* < 0.00001). High statistical heterogeneity was identified across these trials (*I* ^2^ = 70%). Leave‐one‐out sensitivity analysis was done, but no source of heterogeneity could be identified (Figure 3 and Supporting Figure 2).

**FIGURE 3:**
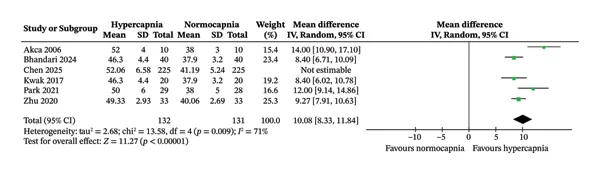
Forest plot comparing the effect of mild hypercapnia versus normocapnia on arterial partial pressure of carbon dioxide (PaCO_2_).

#### 3.3.3. ETCO_2_

Three trials captured ETCO_2_ (mmHg) trends in 117 total patients [10–12]. Mild hypercapnia resulted in a significant rise in ETCO_2_ values (MD = 10.83 mmHg; 95% CI [7.91, 13.76]; *p* < 0.00001). Substantial statistical heterogeneity was noted (*I* ^2^ = 89%). Sensitivity analysis showed that excluding Park et al. [12] eliminated heterogeneity (Figure 4 and Supporting Figure 3).

**FIGURE 4:**
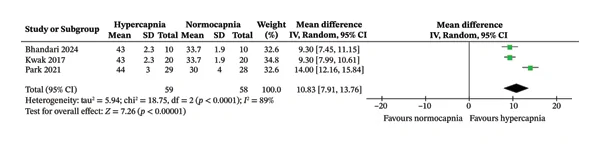
Forest plot comparing the effect of mild hypercapnia versus normocapnia on end‐tidal carbon dioxide (EtCO_2_).

#### 3.3.4. Partial Pressure of Arterial Oxygen (PaO_2_)

Six trials reported data on PaO_2_ (mmHg) across 343 patients [1, 9–13]. No statistically significant difference in PaO_2_ values (MD = −5.57 mmHg; 95% CI: [–18.69, 7.55]; *p* = 0.41) was observed between the treatment arms. Heterogeneity was low among the pooled trials (*I* ^2^ = 13%) (Figure 5).

**FIGURE 5:**
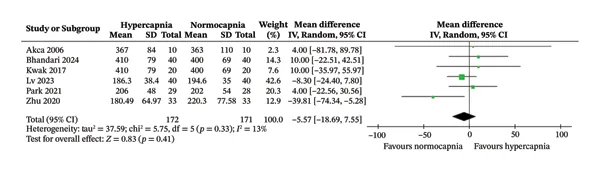
Forest plot comparing the effect of mild hypercapnia versus normocapnia on arterial partial pressure of oxygen (PaO_2_).

#### 3.3.5. Dead Space Fraction (Vd/Vt)

Three trials measured changes in pulmonary dead space fraction in 117 patients [10–12]. Mild hypercapnia was associated with a significant reduction in Vd/Vt percentage compared to normocapnia (MD = −6.38%; 95% CI: [–9.26, −3.50], *p* < 0.00001). No heterogeneity was observed (*I* ^2^ = 0%) (Figure 6).

**FIGURE 6:**
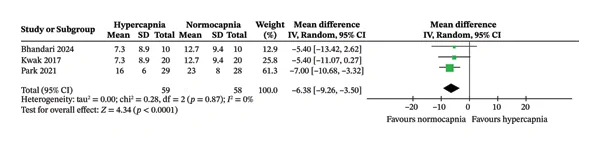
Forest plot comparing the effect of mild hypercapnia versus normocapnia on physiological dead‐space fraction (Vd/Vt).

#### 3.3.6. Arterial pH

Six trials documented arterial blood pH fluctuations among 713 patients in total [7, 9–13]. Patients assigned to the mild hypercapnia group experienced a significant reduction in arterial pH compared with controls (MD = −0.08; 95% CI: [–0.09, −0.07]; *p* < 0.00001). Moderate heterogeneity was found (I^2^ = 35%). Sensitivity analysis demonstrated that excluding Park et al. [12] eliminated heterogeneity (Figure 7 and Supporting Figure 4).

**FIGURE 7:**
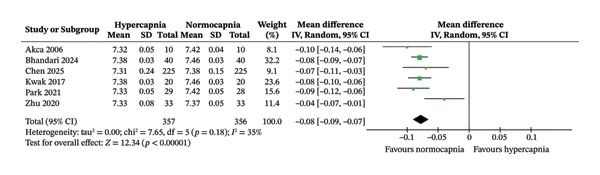
Forest plot comparing the effect of mild hypercapnia versus normocapnia on arterial pH.

#### 3.3.7. MAP

Two trials evaluated intraoperative MAP (mmHg) across 86 patients [9, 13]. No significant difference in MAP between the two ventilation targets (MD = −0.04 mmHg; 95%CI: [–2.03, 1.96]; *p* = 0.97; *I* ^2^ = 0%) was observed in our pooled analysis (Figure 8).

**FIGURE 8:**
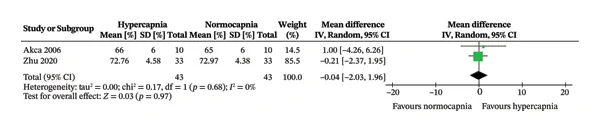
Forest plot comparing the effect of mild hypercapnia versus normocapnia on mean arterial pressure (MAP).

#### 3.3.8. HR

Two trials reported intraoperative HR (bpm) across 516 patients [7, 13]. HR was comparable between the two arms (MD = 0.14 bpm; 95%CI: [–1.00, 1.28]; *p* = 0.81; *I* ^2^ = 0%) (Figure 9).

**FIGURE 9:**
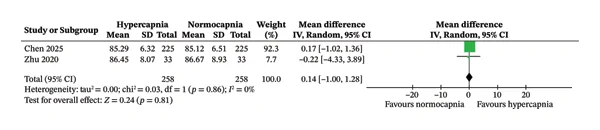
Forest plot comparing the effect of mild hypercapnia versus normocapnia on heart rate (HR).

#### 3.3.9. Subgroup Analyses

We performed subgroup analyses of rSO_2_, PaCO_2_, ETCO_2_, PaO_2_, and arterial pH, according to the type of surgery, characterized as cardiac and noncardiac surgery. However, subgroup analyses did not reduce heterogeneity for any of these outcomes, and no significant differences were observed between the subgroups (Supporting Figures 5, 6, 7, 8, and 9).

### 3.4. Risk of Bias Assessment

Overall, all the RCTs were judged to have moderate risk of bias. Park et al. [12], Kwak et al. [11], and Zhu et al.[13] showed some concerns regarding randomization process as allocation concealment was not described. Lv et al. [1] raised some concerns regarding missing outcome data because eight participants were excluded after randomization, resulting in a modified rather than a true intention‐to‐treat analysis. Some concerns regarding deviations from intended interventions were identified in Lv et al. [1], Park et al. [12], and Bhandari et al. [10] because the anesthesiologists delivering the intervention were aware of group allocation, and no information was provided to control for this lack of blinding. All studies except Lv et al. [1] showed some concerns regarding selective reporting because a prespecified statistical analysis plan was not reported (Table 2).

**TABLE 2:** Risk of bias assessment of included studies using the Cochrane RoB 2.0 tool.

Study	Randomization process	Deviations from intended interventions	Missing outcome data	Outcome measurement	Selective reporting	Overall RoB
Lv et al. [1]	Low	Some concerns	Some concerns	Low	Low	Some concerns
Park et al. [12]	Some concerns	Some concerns	Low	Low	Some concerns	Some concerns
Kwak et al. [11]	Some concerns	Low	Low	Low	Some concerns	Some concerns
Bhandari et al. [10]	Low	Some concerns	Low	Low	Some concerns	Some concerns
Zhu et al.[13]	Some concerns	Low	Low	Low	Some concerns	Some concerns
Akça et al. [9]	Low	Low	Low	Low	Some concerns	Some concerns

We assessed the risk of bias of the cohort study using the ROBINS‐I tool. Chen et al. [7], the only cohort study included, was judged to have an overall moderate risk of bias, primarily due to confounding arising from nonrandom allocation of ventilation strategy. All other domains were judged to be at low risk of bias (Table 3).

**TABLE 3:** Quality assessment by ROBINS I tool.

Study	Bias due to confounding	Bias due to selection of participants	Bias in classification of interventions	Bias due to deviations from intended interventions	Bias due to missing data	Bias in measurement of outcomes	Bias in selection of the reported result	Overall
Chen et al. [7]	Some concerns	Low	Low	Low	Low	Low	Low	Some concerns

### 3.5. GRADE Assessment

According to the GRADE assessment, the overall certainty of evidence ranged from low to very low. The certainty of evidence was low for rSO_2_, PaO_2_, arterial pH, dead space fraction, and MAP, primarily due to concerns regarding risk of bias. Evidence for PaCO_2_, ETCO_2_, and HR was rated as very low due to concerns regarding inconsistency and imprecision, in addition to risk of bias. We downgraded one point for PaCO_2_ and ETCO_2_ due to substantial heterogeneity. Imprecision was identified in ETCO_2_, MAP, and HR because of 95% CIs crossing the line of no effect, along with limited sample sizes. No concerns regarding indirectness were identified in any of the outcomes. Publication bias could not be assessed as less than 10 studies were available for each outcome (Table 4).

**TABLE 4:** Assessment of the certainty of evidence using the GRADE approach.

Outcome	Studies	No. of participants	ROB	Inconsistency	Indirectness	Imprecision	Publication bias	Overall certainty of evidence
rSO_2_	3 RCTs, 1 cohort	608	Serious	Not serious	Not serious	Not serious	Not evaluated	⨁⨁◯◯
								Low

PaCO_2_	5 RCTs, 1 cohort	713	Serious	Serious	Not serious	Not serious	Not evaluated	⨁◯◯◯
								Very low

ETCO_2_	3 RCTs	117	Serious	Serious	Not serious	Serious	Not evaluated	⨁◯◯◯
								Very low

PaO_2_	6 RCTs	343	Serious	Not serious	Not serious	Serious	Not evaluated	⨁⨁◯◯
								Low

Dead space fraction	3 RCTs	117	Serious	Not Serious	Not serious	Serious	Not evaluated	⨁⨁◯◯
								Low

Arterial pH	5 RCTs, 1 cohort	713	Serious	Not serious	Not serious	Not serious	Not evaluated	⨁⨁◯◯
								Low

Mean arterial pressure	2 RCTs	86	Serious	Not serious	Not serious	Serious	Not evaluated	⨁⨁◯◯
								Low

Heart rate	1 RCT, 1 cohort	516	Serious	Not serious	Not serious	Serious	Not evaluated	⨁◯◯◯
								Very low

## 4. Discussion

In this meta‐analysis, mild permissive hypercapnia significantly improved rSO_2_ and respiratory parameters, while maintaining stable hemodynamic parameters such as HR and MAP. Permissive hypercapnia was also associated with an increase in PaCO_2_ and ETCO_2_, accompanied by a modest reduction in arterial pH and dead space fraction. Qualitative analysis of available studies suggested a potential reduction in the incidence of POCD and POD; however, the evidence was limited and heterogeneous.

rSO_2_ serves as a sensitive marker of cerebral perfusion and is used to monitor cerebral ischemia and hypoxia during surgery. Cerebral hypoxia is diagnosed if rSO_2_ is less than 55% or decreases more than 20% from baseline for more than 1 min [14]. Cerebral hypoxia can lead to neuronal injury, mitochondrial dysfunction, neuroinflammation, and disruption of autoregulation mechanisms, which could potentially translate into POCD and POD, increase the length of hospital stay, and increase the risk of stroke or postoperative morbidity [15, 16].

The significant increase in rSO_2_ is therefore the major finding of this study. This increase is biologically plausible and consistent with the known physiological effects of CO_2_ on cerebral vasodilation. PaCO_2_ is the most potent regulator of cerebrovascular tone [12, 17]. Permissive hypercapnia enhances neuronal nitric oxide (NO) synthase activity, increasing NO production, which eventually leads to vasodilation of cerebral blood vessels. Alternatively, an increase in PaCO_2_ produces a permissive reduction in intracellular pH, and even small changes in pH can significantly affect cerebral vascular smooth muscle cells, causing vasodilation. This increases CBF and ultimately increases delivery of oxygen to neural tissues [17]. Experimental studies demonstrate a linear relationship between CBF and PaCO_2_, with increases in PaCO_2_ levels resulting in much larger changes in CBF [17]. However, the improvement in rSO_2_ should be interpreted as a physiological marker rather than a direct patient‐centered clinical outcome. Thus, even though the observed increase in rSO_2_ supports the physiological effect of hypercapnia on cerebral oxygenation, it does not establish improvement in neurological recovery and long‐term clinical outcomes.

Multiple factors during surgery, such as blood loss, anesthetic agents, pneumoperitoneum, and positive‐pressure ventilation, could compromise cerebral oxygen delivery, which could result in postoperative neuronal damage. POCD has gained a lot of attention in research owing to the poor prognosis, impaired quality of life, and increased mortality associated with it. Previous studies reported that POCD affects 25% to 70% of patients after cardiac surgery, and 23% of older patients undergoing noncardiac surgery [18–20]. This is particularly important in elderly patients who often present with several comorbidities and are more likely to have diminished cerebrovascular reserve [21, 22]. Casati et al. demonstrated improvement in rSO_2_ along with improved neurocognitive outcomes and reduced hospital stay in elderly patients undergoing abdominal surgery [22]. Chen et al. also observed improved rSO_2_ accompanied by a reduced occurrence of POCD in elderly patients undergoing laparoscopic surgery [7]. Similarly, the incidence of POD was also reduced in one study due to the neuroprotective role of increased rSO_2_ [23, 24]. However, Lv et al. found no significant difference in the incidence of POD between the hypercapnic and normocapnic groups [1]. This discrepancy could result from the exclusion of elderly patients at increased risk of developing POD, accompanied by a well‐implemented recovery protocol, and administration of dexamethasone, which has historically been shown to lower the risk of POD after surgeries [25, 26]. However, while our qualitative synthesis of POCD and POD is consistent with previous research, it should be considered as exploratory rather than confirmatory because the evidence was limited and heterogeneous.

As expected, targeted hypercapnia increased PaCO_2_ and ETCO_2_. This was accompanied by a modest decrease in arterial pH, reflecting permissive respiratory acidosis. This was consistent with the expected physiological response to hypercapnia. Wong et al. reported that permissive hypercapnia‐induced acidosis resulted in no clinically significant adverse effects such as cardiac arrhythmias in patients undergoing major elective surgery, but contributed to protective effects by improving tissue perfusion, increasing oxygen delivery through the Bohr effect, and reducing inflammatory responses [6]. Interestingly, Lv et al. reported that hypercapnia maintained cerebral oxygenation without any significant variation in arterial pH [1]. Importantly, no adverse effects attributable to reduced pH were reported in any of the included studies.

Another notable finding was the reduction in the dead‐space fraction, observed in patients managed with hypercapnia, which reflects improved respiratory efficiency. Hypercapnia induces hypoxic pulmonary vasoconstriction that redistributes pulmonary blood flow to better ventilated alveoli and improves ventilation–perfusion matching. Our findings are consistent with those of Park et al. who demonstrated that dead space fraction significantly increased in the normocapnic group, whereas this increase was attenuated in the hypercapnic group due to hypercapnic‐induced pulmonary vasoconstriction [12]. Kwak et al. suggested that in addition to improving ventilation–perfusion matching, anti‐inflammatory effects of hypercapnia also reduced lung injury [11]. However, very few studies evaluated this outcome, and whether this translates into improved clinical outcomes remains uncertain. Furthermore, while hypercapnia appears safe in selected patients across these trials, hypercapnia can cause hypoperfusion and lead to atelectasis, intrapulmonary shunting, and hypoxemia, in patients with impaired pulmonary reserve [11].

Importantly, improved rSO_2_ was achieved without disturbing systemic hemodynamic stability, as neither MAP nor HR differed significantly between the hypercapnia and normocapnia arms. Maintenance of MAP is important because adequate cerebral perfusion depends on both cerebrovascular tone and systemic arterial pressure. Hypercapnia stimulates both the sympathetic and the cardiac vagus nerve, with net cardiovascular response depending upon the balance between the two systems [9, 27]. This co‐activation explains why MAP and HR did not significantly differ with hypercapnia. Similarly, no significant difference in partial pressure of arterial oxygen was observed, further emphasizing that hypercapnia increases cerebral oxygen delivery by influencing CBF and not by increasing systemic oxygenation. This is clinically relevant as it supports the potential role of hypercapnia as a neuroprotective strategy in surgical patients without adversely affecting systemic oxygenation or cardiovascular homeostasis.

Current guidelines on perioperative ventilation strategies emphasize lung‐protective ventilation strategies that optimize gas exchange without causing ventilator‐induced lung injury. While normocapnia remains the standard choice, current evidence shows that permissive hypercapnia improves rSO_2_ and exerts anti‐inflammatory effects on the heart, nervous system, and lungs [1, 7, 10, 11, 13]. However, evidence regarding the effects of hypercapnia on clinically meaningful, patient‐centered outcomes remains limited. Nevertheless, it should be kept in mind that controlled hypercapnia is not a universally applicable strategy, but should be individualized according to the patient’s neurological, pulmonary, cardiovascular, and anesthetic context. Hypercapnia can increase CBF and thus, may be undesirable in patients with impaired intracranial compliance or elevated intracranial pressure [28]. It may also increase pulmonary vascular resistance, placing additional stress on patients with pulmonary hypertension or right ventricular dysfunction [29]. Patients with limited pulmonary reserve or an impaired ability to tolerate respiratory acidosis may likewise require individualized carbon dioxide targets [29]. The results of this meta‐analysis therefore cannot be extrapolated to these high‐risk clinical settings. Large randomized clinical trials and observational studies evaluating patient‐centered clinical outcomes, such as POCD, POD, neurological complications, and long‐term functional recovery, in diverse surgical populations are required before this strategy could be integrated into routine clinical practice. Future research should also focus on identifying patients in whom hypercapnia can provide clinical benefit and in those in whom it may be unsafe.

Our study possesses several strengths. To our knowledge, it is the first systematic review and meta‐analysis to comprehensively evaluate the impact of permissive hypercapnia across diverse surgical populations. By combining evidence across diverse surgical populations, our meta‐analysis provides an overall estimate of the physiological effects of permissive hypercapnia across several surgical settings; however, the clinical heterogeneity limits the extent to which these findings can be generalized across surgical specialties. From a methodological perspective, our systematic review incorporated comprehensive searching, independent duplicate screening and extraction, and validated quality assessment tools. We performed a GRADE quality assessment allowing clinicians to assess the confidence in the effect estimate.

Several limitations are present in the meta‐analysis. First, although the protocol and methodological framework were established prospectively at our institution before study screening commenced, registration was performed retrospectively. Therefore, the absence of prospective registration may limit transparency regarding the prespecification of the review methods. Second, the number of included study participants for various outcomes was relatively small; hence, findings should be interpreted with caution. Third, substantial clinical heterogeneity existed among the included studies with respect to baseline patient characteristics, surgical procedure, anesthetic dose, type and technique, duration of surgery, and targeted carbon dioxide levels, which may have influenced the pooled effect estimates. Permissive hypercapnia is not a uniformly applied intervention, and its physiological effects may vary according to the degree and duration of hypercapnia, baseline pulmonary and cardiovascular function, cerebral autoregulatory capacity, intracranial compliance, and perioperative management. Although subgroup analyses according to cardiac versus noncardiac surgery were performed for several outcomes, these analyses did not eliminate heterogeneity or demonstrate significant differences between subgroups. The limited number of studies prevented more comprehensive analyses according to age, specific surgical procedures, baseline characteristics, anesthetic technique, or hypercapnia target. Fourth, postoperative cognitive outcomes could not be evaluated quantitatively due to the limited number of studies and variations in assessment tools and follow‐up duration. Fifth, the included evidence had methodological limitations. All RCTs had at least some concerns regarding risk of bias, while the single observational cohort was judged to have moderate overall risk of bias, primarily because of confounding related to nonrandom allocation of the ventilation strategy. Sixth, the GRADE assessment rated the certainty of evidence as low to very low across all outcomes, limiting confidence in the pooled estimates and preventing definite conclusions regarding the observed benefits of permissive hypercapnia. Therefore, our findings, especially regarding rSO_2_, should be considered as exploratory rather than definitive. Finally, we were limited in our ability to formally assess publication bias as fewer than 10 studies were included for each outcome.

## 5. Conclusion

In conclusion, this systematic review and meta‐analysis demonstrated that permissive hypercapnia significantly improved intraoperative regional cerebral oxygenation (rSO_2_) compared with normocapnia in patients undergoing surgical procedures, but the available evidence does not establish that this physiological improvement translates into improved neurocognitive or long‐term clinical outcomes. No significant adverse effects on HR and MAP were observed, which supports hemodynamic stability. However, substantial clinical heterogeneity, methodological limitations, and low to very low certainty of evidence limit the strength and generalizability of these findings. Thus, adequately powered RCTs focused especially on standardized hypercapnia targets and patient‐centered neurological outcomes are needed before routine implementation can be recommended.

## Author Contributions

Areej Riaz conceived and supervised the study, participated in data extraction, provided methodological guidance, drafted the manuscript, and critically reviewed and edited the manuscript throughout all stages of the project. Adeel Ahmed Khan conducted the literature search, study screening, data extraction, formal data analysis, interpretation of results, reference management, and manuscript drafting. Fatima Nawaz contributed to study screening, data analysis, interpretation of results, and manuscript revision. Amna Shafaqat contributed to the study methodology and data extraction. Amna Khan drafted the Introduction section, while Hadia Sheraz drafted the Discussion section. Mohammed M. AlTelbany, Nauroze Khan, and Bismah Rafique Malik contributed to study screening. Hermann Yokolo served as the senior supervisor, providing overall scientific oversight, methodological guidance, and critical review of the manuscript. All authors contributed to the study.

## Funding

This research did not receive any specific grant from funding agencies in the public, commercial, or not‐for‐profit sectors.

## Disclosure

All authors approved the final version of the manuscript and agreed to be accountable for all aspects of the work.

## Conflicts of Interest

The authors declare no conflicts of interest.

## Supporting Information

Additional supporting information can be found online in the Supporting Information section.

## Supporting information


**Supporting Information 1** Supporting File 1: Forest plots illustrating the results of sensitivity and subgroup analyses, along with the complete electronic search strategies used to identify eligible studies across the searched databases.

**Supporting Information 2** PRISMA 2020 Checklist.

## Data Availability

The data supporting the findings of this study are available from the corresponding author upon reasonable request.
