# Supplementary material for: Effects of Permissive Hypercapnia Versus Normocapnia on Cerebral Oxygenation and Postoperative Outcomes in Adult Surgical Patients: A Systematic Review and Meta‐Analysis
Source: Anesthesiol Res Pract. 2026 Oct 31;2026:3569464. doi: 10.1155/anrp/3569464 (PMC13723312; doi:10.1155/anrp/3569464)
Supplement: Supplementary file 1 [file ANRP-2026-3569464-s001.docx]

**Effects of Permissive Hypercapnia versus Normocapnia on Cerebral Oxygenation and Postoperative Outcomes in Adult Surgical Patients: A Systematic Review and Meta-analysis**

Areej Riaz, Amna Khan, Fatima Nawaz, Amna Shafaqat, Hadia Sheraz , Mohammed M ALTelbany, Nauroze Khan, Adeel Ahmed Khan, Bismah Rafique Malik, Hermann YOKOLO

*Anesthesiology Research and Practice*

**CORRESPONDING AUTHOR**

Hermann YOKOLO

Department of research, Medical Research Circle (MedReC), Goma, DR Congo

Kyeshero Lusaka street 218, Postal code 73 Gisenyi, Goma, Nord Kivu, DR Congo

[hermannyokolo40@outlook.com](mailto:hermannyokolo40@outlook.com)

<https://orcid.org/0009-0002-8347-7236>

**Supplementary Figure 1.** Leave-one-out sensitivity analysis of the effect of mild hypercapnia versus normocapnia on intraoperative regional cerebral oxygen saturation (rSO₂).

**Supplementary Figure 2.** Leave-one-out sensitivity analysis of the effect of mild hypercapnia versus normocapnia on arterial partial pressure of carbon dioxide (PaCO₂).

**Supplementary Figure 3.** Leave-one-out sensitivity analysis of the effect of mild hypercapnia versus normocapnia on end-tidal carbon dioxide (EtCO₂).

**Supplementary Figure 4.** Leave-one-out sensitivity analysis of the effect of mild hypercapnia versus normocapnia on arterial pH.

**Supplementary Figure 5.** Subgroup analysis of the effect of mild hypercapnia versus normocapnia on intraoperative regional cerebral oxygen saturation (rSO₂) based on the type of surgery.

**Supplementary Figure 6.** Subgroup analysis of the effect of mild hypercapnia versus normocapnia on arterial partial pressure of carbon dioxide (PaCO₂) based on the type of surgery.

**Supplementary Figure 7.** Subgroup analysis of the effect of mild hypercapnia versus normocapnia on end-tidal carbon dioxide (EtCO₂) based on the type of surgery.

**Supplementary Figure 8.** Subgroup analysis of the effect of mild hypercapnia versus normocapnia on arterial partial pressure of oxygen (PaO₂) based on the type of surgery.

**Supplementary Figure 9.** Subgroup analysis of the effect of mild hypercapnia versus normocapnia on arterial pH based on the type of surgery.

**Search String:**

("Hypercapnia"[Mesh] OR hypercapnia*[tiab] OR hypercarbia*[tiab] OR "Carbon Dioxide"[Mesh] OR "Carbon Dioxide"[tiab] OR carbon dioxide[tiab] OR CO2[tiab] OR PaCO2[tiab] OR PaCO₂[tiab] OR "arterial carbon dioxide"[tiab] OR "arterial carbon dioxide tension"[tiab] OR "arterial partial pressure of carbon dioxide"[tiab] OR "partial pressure of carbon dioxide"[tiab] OR "arterial carbon dioxide pressure"[tiab] OR EtCO2[tiab] OR EtCO₂[tiab] OR PETCO2[tiab] OR PETCO₂[tiab] OR "end tidal carbon dioxide"[tiab] OR "end-tidal carbon dioxide"[tiab] OR "end tidal CO2"[tiab] OR "end-tidal CO2"[tiab] OR "end expiratory carbon dioxide"[tiab] OR capnograph*[tiab] OR "Capnography"[Mesh]) AND ("Surgical Procedures, Operative"[Mesh] OR surg*[tiab] OR operation*[tiab] OR operative[tiab] OR intraoperative[tiab] OR perioperative[tiab] OR postoperative[tiab] OR anesthesia[tiab] OR anaesthesia[tiab] OR anesthetic*[tiab] OR anaesthetic*[tiab] OR "Anesthesia"[Mesh]) AND ("Near-Infrared Spectroscopy"[Mesh] OR "Oximetry"[Mesh] OR "Monitoring, Intraoperative"[Mesh] OR NIRS[tiab] OR "near infrared spectroscopy"[tiab] OR "near-infrared spectroscopy"[tiab] OR rSO2[tiab] OR rSO₂[tiab] OR ScO2[tiab] OR ScO₂[tiab] OR "regional cerebral oxygen saturation"[tiab] OR "cerebral oxygen saturation"[tiab] OR "brain oxygenation"[tiab] OR "cerebral oxygenation"[tiab] OR "cerebral tissue oxygenation"[tiab] OR "brain tissue oxygenation"[tiab] OR "cerebral tissue oxygen saturation"[tiab] OR "cerebral oximetry"[tiab] OR "brain oximetry"[tiab])
